# Hepatitis A Virus Vaccine Escape Variants and Potential New Serotype Emergence

**DOI:** 10.3201/eid1704.101169

**Published:** 2011-04

**Authors:** Unai Pérez-Sautu, M. Isabel Costafreda, Joan Caylà, Cecilia Tortajada, Josep Lite, Albert Bosch, Rosa M. Pintó

**Affiliations:** Author affiliations: University of Barcelona, Barcelona, Spain (U. Pérez-Sautu, M.I. Costafreda, A. Bosch, R.M. Pintó);; Public Health Agency of Barcelona, Barcelona (J. Caylà, C. Tortajada);; CatLab, Viladecavalls, Spain (J. Lite)

**Keywords:** viruses, hepatitis A virus, molecular epidemiology, quasispecies, capsid structural constraints, codon usage, rare codon, vaccine escape variants, vaccine escape mutants, dispatch

## Abstract

Six hepatitis A virus antigenic variants that likely escaped the protective effect of available vaccines were isolated, mostly from men who have sex with men. The need to complete the proper vaccination schedules is critical, particularly in the immunocompromised population, to prevent the emergence of vaccine-escaping variants.

In areas where hepatitis A has low to moderate endemicity, introduction of the virus occurs through consumption of imported foods, traveling, or through immigration flows (*1**–**3*). Men who have sex with men (MSM) comprise a high-risk group for hepatitis A, and several outbreaks affecting this group have been reported across Europe (*4*). To prevent the spread of infection, since 1999, vaccination programs have been implemented among preadolescents in the Catalonia Autonomous Community of Spain.

Despite some degree of nucleotide heterogeneity at the capsid region of hepatitis A virus (HAV) (*5**,**6*), there is not an equivalent degree of amino acid variation (*7*). HAV replicates as complex dynamic mutant distributions or quasispecies (*8*) and thus the high degree of conservation of the capsid amino acid sequences among independent strains must be the result of negative selection on newly arising mutants. So far, a single serotype of human HAV has been recognized, which suggests that severe structural constraints occur in the capsid that prevent the more extensive substitutions necessary for the emergence of a new serotype. Indeed, negative selection of replacements affecting residues encoded by rare codons of the capsid surface has been documented, indicating a critical role played by such rare codons (*9*). Since these residues are located quite near or even at the epitope regions, the need to maintain such rare codons might prevent the emergence of new serotypes (*9*). We have recently noted that fine-tuning translation kinetics selection, or the right combination of preferred and rare codons in the capsid coding region, is necessary to get regulated ribosome traffic to guarantee the proper capsid folding (*10*). In this context, it seems quite unlikely that a new serotype will emerge, although the emergence of new variants is not impossible if the virus population is forced through bottleneck conditions such as immune selective pressures. We investigated the presence of antigenic variants among sporadic and outbreak cases of hepatitis A.

## The Study

**Figure 1:**
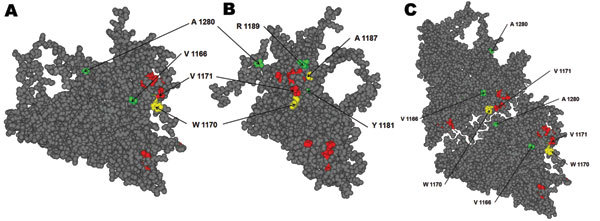
Hepatis A virus protomer model (11; refined by Ming Luo, University of Alabama, Birmingham, AL, USA), which includes the locations of all of the substituted residues in viral protein 1 detected in the isolated variants during 2005–2009. A) Front view of the external surface. B) Lateral view. C) View of 2 adjacent protomers, showing the close contact of residues 1171 and 1280. Red, residues forming the immunodominant site; yellow, residues substituted in monoclonal antibody–resistant mutants C6 (W1170C) and P29 (A1187P); green, residues substituted in the identified natural variants. The amino acid substitution V1171A detected in 1 variant is shown in red because this residue belongs to the immunodominant site.

We molecularly characterized 128 HAV strains isolated during 2005–2009 in Catalonia from patients with both sporadic (n = 37) and outbreak (n = 91) cases (Technical Appendix Figure 1) based on their viral protein 1 (VP1) region (*7*). Deduced amino acid sequences were compared with those of HM-175 and GBM strains (GenBank accession nos. M14707 and X75215, respectively) and constituents of 2 of the commercial HAV vaccines, HAVRIX (GlaxoSmithKline, Rixensart, Belgium) and Avaxim (Sanofi-Pasteur, Paris, France), respectively. Six amino acid replacements, which have not been previously described, were detected (Table 1). Two were semiconservative replacements, V1171A and A1280V, and the other 4 were nonconservative, V1166G, Y1181S, R1189T, and A1280E. The replaced amino acids were located in a refined 3-dimensional computer model of the HAV protomer (*11*), and their relative distances to residues 1102, 1171 and 1176, constituents of the immunodominant site (*12*), and to residue 1221, constituent of the glycophorin A binding site epitope (*13*), were used as markers of the potential antibody-escaping phenotype. All replaced positions were located at (1171) or around (1166, 1181, 1189, 1280) the viral immunodominant site near the 5-fold axis (Figure 1), and thus strains bearing these replacements might be considered antigenic variants. In a previous study, several escape mutants to K34C8 monoclonal antibody (MAb), which recognizes the immunodominant site, were isolated (*9*). Among these mutants, 2 were defined by replacements W1170C (C6) and A1187P (P29), which were located very close to the mutated residues detected in this study (Figure 1). Residue 1170 is located contiguous to residue 1171 and close to residues 1280 and 1181. Additionally, residue 1187 is in close contact with residue 1189. Since HAV natural isolates cannot be grown in vitro, C6 and P29 monoclonal antibody–resistant (MAR) mutants were used to mimic the behavior of the naturally isolated variants in neutralization assays with antivaccine serum specimens. Results proved that mutant C6 is resistant to both antivaccine serum, as well to convalescent-phase serum, whereas mutant P29 is partially resistant to serum generated with Avaxim vaccine (Table 2).

**Table 1:** Amino acid substitutions in the VP1 protein observed in different strains isolated during the present study*

Strain	Replacement	Position*
MSM08-09-219	V → G	1166
MSM08-09-186	V → G	1166
	V → A	1171
BCN60	Y → S	1181
MSM08-09-ClusterE	R → T	1189
MSM08-09-144	A → V	1280
BCN31	A → E	1280

*Position strain HM175 (GenBank accession no. M14707). The first digit refers to the viral protein, i.e., 1 for VP1, and the following 3 digits refer to the amino acid position in the protein.

**Table 2:** Neutralization assays of K34C8 MAb-escape variants that showed replacements at the same or very close positions as the mutated positions in the naturally-selected field variants isolated during 2005–2009*†

Mutant (position replaced)	log N_t_/N_0_ vaccine serum (HAVRIX)	log N_t_/N_0_ vaccine serum (Avaxim)	log N_t_/N_0_ convalescent-phase serum (HCS2)	log N_t_/N_0_ MAb K34C8
C6 (1170)	–0.08 ± 0.14	–0.08 ± 0.14	–0.02 ± 0.04	–0.08 ± 0.14
P29 (1187)	–0.70 ± 0.09	–0.30 ± 0.19	–0.70 ± 0.07	–0.37 ± 0.19
D23 (1217)	–0.88 ± 0.02	–0.54 ± 0.01	–0.61 ± 0.07	–0.58 ± 0.12
HM175/43c	–0.69 ± 0.09	–0.60 ± 0.05	–0.65 ± 0.05	–0.61 ± 0.10

*Assays were performed by using vaccine and convalescent-phase serum samples as well as K34C8 MAb. MAb, monoclonal antibody; HAVRIX, HAVRIX vaccine (GlaxoSmithKline, Rixenart, Belgium); Avaxim, Avaxim vaccine (Sanofi-Pasteur, Paris, France). †Following the model of the hepatitis A virus protomer of Ming Luo (Figure 1). Three neutralization assays were performed with each antivaccine serum sample, the convalescent-phase serum sample, and the MAb K34C8. As controls, neutralization of the D23 H7C27 MAb escape variant (*9*) as well as that of the HM175/43c wild-type strain, was also measured. The highest dilution showing a log N_t_/N_0_ = –0.60 (75% neutralization) of the wild-type strain was used to test the variants; N_t_, the viral titer after neutralization; N_0_, the initial titer. Neutralization limits were the following: log Nt/N_0_>–0.26 (<45%) for resistant variants, –0.26>log Nt/N_0_>–0.60 (45%–75%) for partially resistant variants, and log Nt/N_0_<–0.60 (>75%) for sensitive variants (*9*).

**Figure 2:**
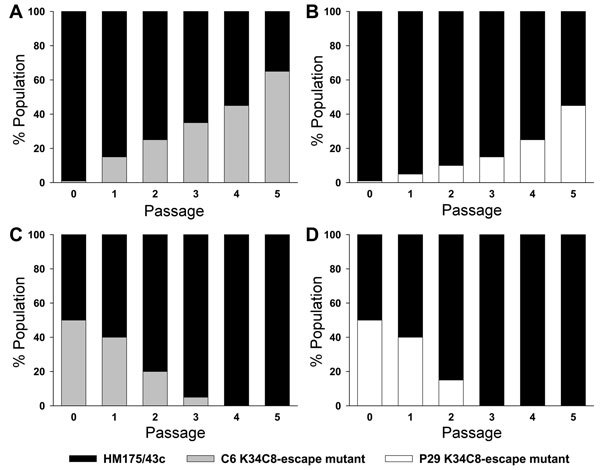
Growth competition experiments. Monoclonal antibody–resistant (MAR) mutants C6 (W1170C) and P29 (A1187P) were grown in competition with the HM175/43c (wild-type virus) in the presence (A, B) or in the absence (C, D) of the monoclonal antibody (MAb) K34C8. The MAR/wild-type ratios were 1:100 (104 50% tissue culture infective dose [TCID50] units of MAR mutants mixed with 106 TCID50 units of the wild-type virus in the presence of the K34C8 MAb) and 1:1 (106 TCID50 units of MAR mutants mixed with 106 TCID50 units of the wild-type virus in the absence of antibodies). In the competition experiments performed in the presence of antibodies, the initial viral mixtures as well as the viral progenies were neutralized with the MAb prior each infection passage. The proportion of mutant and wild-type phenotypes at each passage was inferred from the chromatogram of the consensus sequences and using as marker mutations W1170C and A1187P in C6 and P29 MARs, respectively (*9*).

Of the 6 antigenic variants isolated (Table 1), 4 were obtained during an outbreak among MSM in 2008–2009. Although the number of reported cases of this outbreak was of 186, the number of molecularly analyzed samples, i.e., 66, was similar to that of the rest of analyzed samples, i.e., 62 (including other small outbreaks as well as sporadic cases). Phylogenetic analysis of antigenic variants in the MSM group suggested that they originated from a single patient (Technical Appendix Figures 1, 2), and thus 4 variants (6% of all isolated strains) represent quite an important number for such an antigenically stable virus. In contrast, the multiple origins of the strains of the general group (42 phylogenetically distinguishable strains; Technical Appendix Figure 1) did not correlate with higher numbers of antigenic variants: only 2 were detected (3% of all isolated strains). An intriguing issue is why so many variants of the immunodominant 5-fold site arose during the MSM 2008-2009 outbreak, considering the low fitness shown by MAR mutants with replacements around this site. In particular, C6 and P29 mutants were rapidly outcompeted by a wild-type strain in the absence of antibodies, and although they were able to overcome the wild-type virus in the presence of the K34C8 MAb, it was only after a slow process (Figure 2), indeed indicating a very low fitness. This kind of mutant can only be selected throughout bottleneck events, such as the ingestion of minute amounts of viruses able to float some variants, unlikely in high-risk practices of HIV-positive patients whose viral load in stool may be as high as 10^11^ genome copies/g, or the ingestion of a considerable amount of viruses by patients with low IgG levels, who are unable to completely neutralize the infecting virus, thus allowing the viral population to replicate in the presence of antibodies. In fact, 4% (8/186 case-patients) of the MSM 2008–2009 outbreak patients had been vaccinated. However, in only 1 case was the vaccine administered during childhood following the complete dose schedule. In 5 cases, the patients had received only 1 dose of the vaccine during the 6-month period before any symptoms of infection, and the remaining 2 had received only 1 dose of the vaccine long before the infection. Five of these 8 patients were HIV positive. An incomplete vaccination schedule in an immunocompromised host could lead to a situation of only partial protection, providing suitable conditions for the emergence of an antigenic variant. Unfortunately, samples from these vaccinated patients were not available; however, it is obvious that the vaccinees likely contributed to the selection of such antigenic variants at a population scale. Because in situations of no competition with the wild-type virus, the MAR mutants replicate perfectly well, it may be inferred that the natural variants, once they are selected in an improperly vaccinated HIV-positive person, may spread to other, properly vaccinated persons.

## Conclusions

Isolation of so many variants in a single outbreak among the MSM population, in a virus presenting such severe genomic and structural constraints, emphasizes the need to target this community with more effective information on risky sexual practices and vaccination programs. Additionally, and particularly among HIV-positive MSM, efforts should be made to completely accomplish the vaccination schedule, due to their lower level of immune response (*14**,**15*). An additional concern is that this impaired response may contribute not only to a lower protection of the vaccinee but also to the emergence of antigenic variants. In the analyzed MSM 2008–2009 outbreak, 4 variants were isolated that were located at or very close to the immunodominant site as well as to residues substituted in 2 MAR mutants showing a phenotype of resistance to the protection offered by commercial vaccines. Thus, a similar behavior of the natural variants can be postulated, and if this is the case, a new serotype could emerge.

## Supplementary Material

Technical Appendix Phylogenetic tree figure and temporal distribution of stain isolation figure.
