# Supplementary material for: Hepatitis A Virus Vaccine Escape Variants and Potential New Serotype Emergence
Source: Emerg Infect Dis. 2011 Apr;17(4):734–7. doi: 10.3201/eid1704.101169 (PMC3377408; doi:10.3201/eid1704.101169)
Supplement: Technical Appendix — Phylogenetic tree figure and temporal distribution of stain isolation figure. [file 10-1169-Techapp-s1.pdf]

Article DOI: 10.3201/eid1704.10.1169

Hepatitis A Virus Vaccine Escape Variants and Potential New Serotype Emergence

Technical Appendix

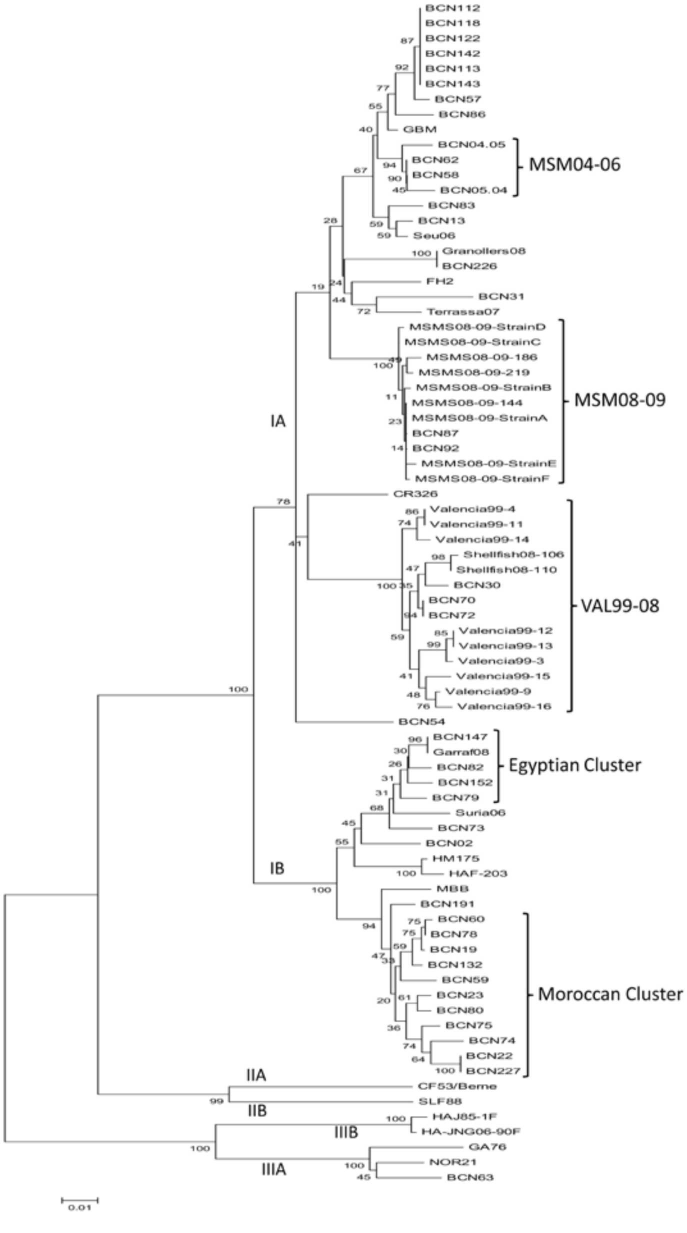

Technical Appendix Figure 1. Phylogenetic tree based on the C-terminal fragment of viral protein 1. Neighbor-joining, Kimura 2-parameter, bootstrap 1,000 replicates (GenBank accession nos: HQ401214–HQ401267).187 × 252 mm.

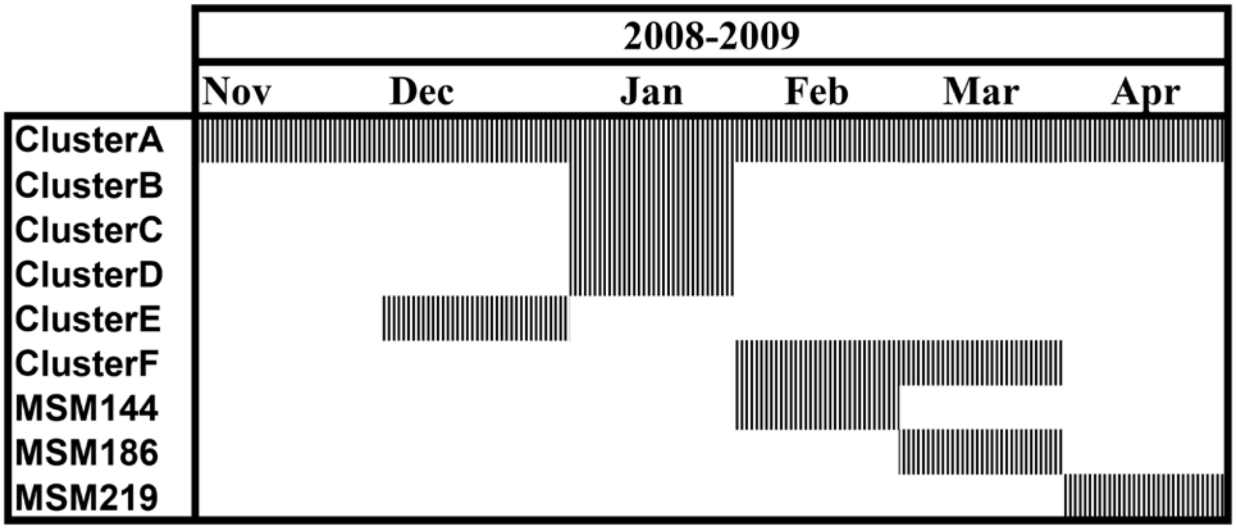

Technical Appendix Figure 2. Temporal distribution of strain isolation during the 2008–2009 hepatitis outbreak among men who have sex with men (MSM). Nine distinguishable strains were isolated. Cluster E and strains MSM08-09-144, MSM08-09-186, and MSM08-09-219 are antigenic variants that evolved from the original and most abundant cluster A. 189 ×81 mm.
